# Diverse role of NMDA receptors for dendritic integration of neural dynamics

**DOI:** 10.1371/journal.pcbi.1011019

**Published:** 2023-04-10

**Authors:** Yuanhong Tang, Xingyu Zhang, Lingling An, Zhaofei Yu, Jian K. Liu

**Affiliations:** 1 Institute for Artificial Intelligence, Department of Computer Science and Technology, Peking University, Beijing, China; 2 Guangzhou Institute of Technology, Xidian University, Guangzhou, China; 3 School of Computer Science and Technology, Xidian University, Xi’an, China; 4 School of Computing, University of Leeds, Leeds, United Kingdom; Chinese Academy of Sciences, CHINA

## Abstract

Neurons, represented as a tree structure of morphology, have various distinguished branches of dendrites. Different types of synaptic receptors distributed over dendrites are responsible for receiving inputs from other neurons. NMDA receptors (NMDARs) are expressed as excitatory units, and play a key physiological role in synaptic function. Although NMDARs are widely expressed in most types of neurons, they play a different role in the cerebellar Purkinje cells (PCs). Utilizing a computational PC model with detailed dendritic morphology, we explored the role of NMDARs at different parts of dendritic branches and regions. We found somatic responses can switch from silent, to simple spikes and complex spikes, depending on specific dendritic branches. Detailed examination of the dendrites regarding their diameters and distance to soma revealed diverse response patterns, yet explain two firing modes, simple and complex spike. Taken together, these results suggest that NMDARs play an important role in controlling excitability sensitivity while taking into account the factor of dendritic properties. Given the complexity of neural morphology varying in cell types, our work suggests that the functional role of NMDARs is not stereotyped but highly interwoven with local properties of neuronal structure.

## Author summary

A single neuron receives a large number of inputs from its dendrites. A fundamental principle for neural dynamics is how these inputs are collectively integrated and converged to the neuronal soma to generate diverse patterns of action potentials. Such a principle has been formulated as a question of dendritic integration, which depends on the biophysical properties of neuronal dendrites and their associated synapses. It has been documented that NMDA plays an important role in dendritic integration. However, it remains unclear how dendritic integration is related to the distribution of NMDA receptors on various parts of the dendritic morphology of a neuron. Here we use Purkinje cell as a model system to symmetrically investigate this question, as the Purkinje cell exhibits a mixture of simple and complex spikes. By exploring the role of NMDA at different parts of dendritic branches and regions, we found somatic responses are diverse from simple to complex spikes, depending on specific dendritic branches. Our results suggest that the functional role of NMDA is highly interplayed with the local properties of neuronal dendrites.

## 1 Introduction

One of the ubiquitous features of neurons is that there is a complex and diverse structure of dendrites converging to the neural soma [1]. The connections between neurons are distributed over dendrites and manifested by different types of synaptic receptors [2]. The NMDA (N-methyl-D-aspartate) receptor (NMDAR) is a common receptor-gated glutamate channel that regulates the release of presynaptic neurotransmitters and participates in postsynaptic responses, playing a crucial role in modulating synaptic transmission in various types of neurons [3–12]. The cerebellum plays an important role in many types of cognitive behaviors in the brain function [13, 14]. In the cerebellum, the functional role of NMDAR has been studied over years. Existing studies have found that postsynaptic NMDARs carry part of the climbing fiber (CF) mediated excitatory postsynaptic current in Purkinje cells (PCs) [15], which was almost undetectable in the first month after mice birth, and reached mature expression at about 2 months old [16]. Moreover, NMDAR expression in CF-PC synapses promotes spontaneous complex spike (CS) activity by enhancing endogenous current and after-hyperpolarization current induced by CS [17]. By applying the NMDAR blocker in mature rat PCs, it was demonstrated that NMDARs play a similar role in the synaptic plasticity of parallel fibers (PFs) to PCs as it does in CFs [18]. As a result, over-expressed NMDARs at the PF-PC synapse deteriorated cerebellar plasticity and motor learning [19, 20].

According to the classical view of cerebellar physiology, NMDAR plays a limited role in the activity of PCs via PFs from granule cells [16, 18, 19, 21]. However, other evidence suggested that NMDARs could be involved in the PC activity via PFs modulated by climbing fibers from the inferior olivary nucleus [16, 18, 22, 23]. These experimental observations raise a debate about how NMDAR contributes to regulating the dynamics of PF-PC synapses [24, 25]. Furthermore, some studies considered the distribution and functional characteristics of NMDARs [26, 27], and suggested that the location of NMDARs on dendrites may be the decisive factor of their signal transmission ability [28]. The branch-specific response can increase dendritic computing power for neurons, and the signal actively integrated into soma can effectively improve the information processing power of PC dendrites [29, 30]. By triggering local dendritic peaks at different PF input intensities, individual PCs can shift between linear and burst-pause encoders to implement branch-dependent multiplexed coding to increase cerebellar coding and learning capacity [31–33]. Synapse formation between PF and PC is very abundant, and these PF-PC synapses form various forms of plasticity [15, 34]. These studies suggest that the dendritic characteristics of PCs, especially synaptic location, distribution, and number could be a key factor for the function of PCs [18].

The current research on NMDARs in the PC circuit mainly focuses on their role in plasticity expression [18, 21, 26]. However, it has been found that NMDARs can inhibit the spontaneous simple spike (SS) discharge of PC by increasing the excitability of intermediate molecular neurons [27], and the tetanic activation of NMDA receptor outside the synapse can reduce intrinsic excitability, promote bistable activity, and ultimately affect neuron discharge [35]. Furthermore, for the synaptic dynamics in neurons, NDMARs are slower, compared to AMPA receptors, to respond to incoming spikes. This long-duration process of NMDARs controls the range of signal events and is essential for synaptic function as it acts as a storage device for associating rapid presynaptic signals with longer postsynaptic signals for neural dynamics [36]. Therefore, it is necessary to study the role of NMDARs in regulating synaptic input on PCs for information integration. In particular, it is highly desirable to consider a detailed examination of the distribution of NMDARs in the complex dendritic tree of PCS and their effect on the dendritic and somatic response.

Here, utilizing a detailed PC model with dendritic morphology, we investigate the role of NMDARs in dendritic and somatic response at different parts of dendritic branches and regions. We demonstrate that there are diverse response patterns induced by NMDARs depending on their locations. Using the same input, somatic response patterns can switch from silent, to simple spikes and complex spikes, depending on which specific dendritic branch is triggered. When the number of NMDARs increases, more spikes are induced at some specific branches, but strikingly, spikes are also quite conserved and do not change at some other branches. Our detailed examination of the dendrites regarding their diameters and distance to soma reveals diverse response patterns, yet explains two firing modes, simple and complex spikes of PCs. Taken together, these results suggest that NMDARs play an important role in controlling excitability sensitivity while taking into account the factor of dendritic properties. Given the complexity of neural morphology varying in cell types, our results suggest that the functional role of NMDAR is not stereotyped but highly interleaved with local properties of neuronal morphology.

## 2 Results

### 2.1 Somatic response depending on the NMDAR dendritic location

Using a detailed PC model with dendritic morphology, we systematically explored how NMDARs regulate somatic response. A simple scenario of synaptic dynamics was considered according to whether there are NMDARs expressed in PC dendrites, where both AMPA and NMDA synapses were modeled with typical fast and slow dynamics, respectively (see Methods).

**Fig 1:**
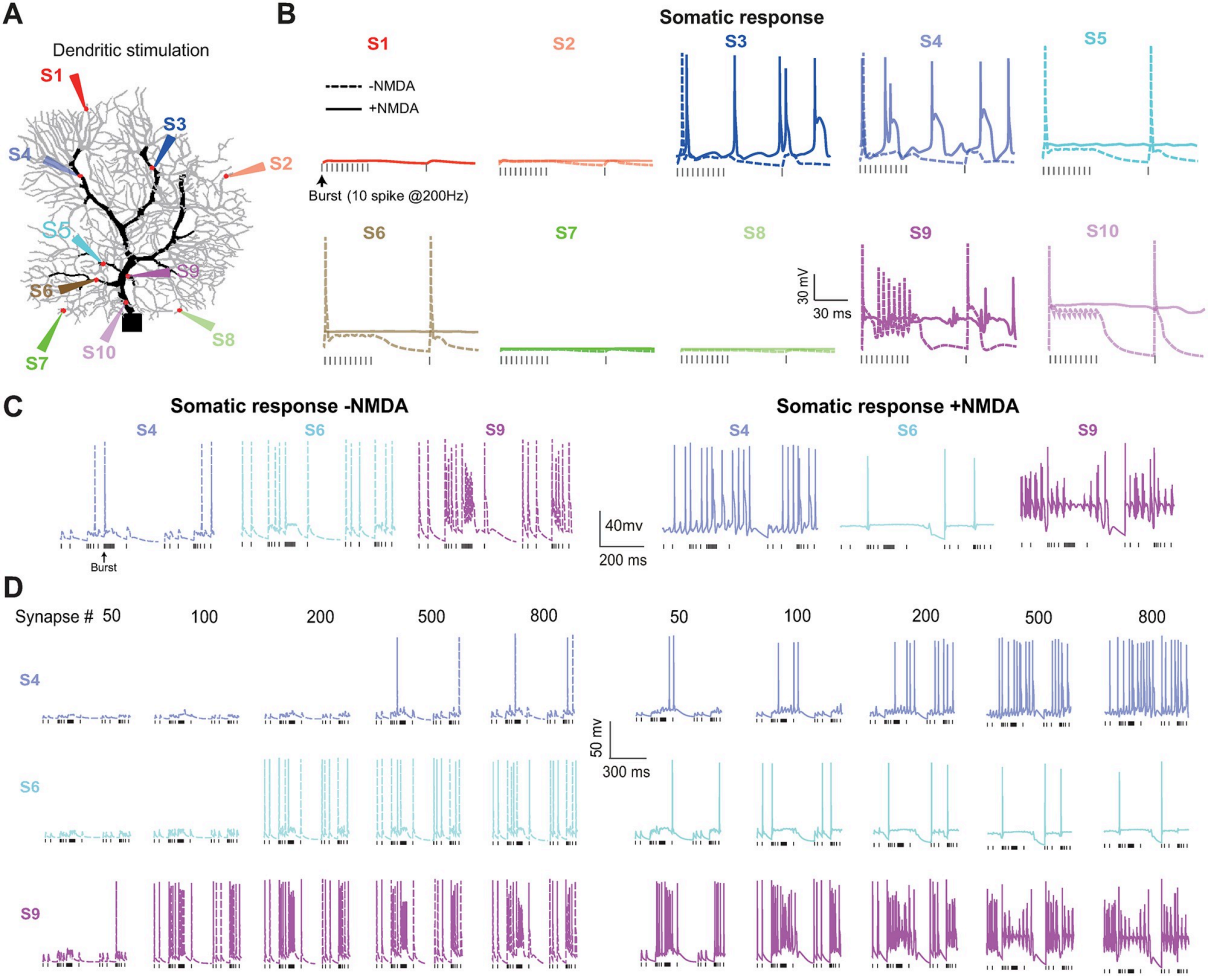
Diverse somatic responses depending on the dendritic location of NMDARs. (A) Stimulating 10 different sites individually on a PC morphological dendritic field. S1–S10 are typical sites, from spiny to primary dendrites. (B) PC somatic responses triggered by each stimulation site with and without NMDARs. 1000 NMDAR synapses were concentrated around each simulation site. (C) Detailed somatic membrane potential traces at three representative sites. S4: increased simple spikes; S6: decreased simple spikes; S9: change of complex spikes. (D) Somatic response profiles with the increasing number of synapses stimulated.

To see the effect of NMDR on somatic response, we distributed 1000 NMDARs around a typical dendritic site and recorded soma membrane potential. The varying length and diameter of dendrites produce different input resistance, which causes diverse degrees of response and affects the somatic spike discharge. According to the morphological and structural characteristics of the modeled PC, we selected 10 typical sites (S1–S10) of dendrites, based on dendrite diameter and distance from the soma, to explore the diversity of PC somatic response induced by different locations of NMDARs (Fig 1A). In terms of distance of dendrite to soma, S10 and S9 are closest to soma, followed by S5 and S6, then S7 and S8, S3 and S4, and finally S1 and S2 are farthest. For the diameter of the dendrite, S10 and S9 are the largest, followed by S4 and S3, then S5 and S6, until the spiny dendrites S7, S8, S1, and S2.

With a background 10 Hz Poisson spikes stimulus first, a sequence of 10 spikes at 200 Hz was injected into each synapse on S1–S10 with or without NMDAR. We found that there is a diverse response profile depending on the dendritic stimulation site (Fig 1B). For the synapses located in spiny dendrites with smaller diameters (S1, S2, S7, and S8), silent somatic responses were found, independent of the existence of NMDARs. For the synapses located at S3 and S4, which are far away from soma yet have larger dendritic diameters, NMDAR significantly enhances the excitability and contributes to generating somatic spikes. However, when the synapses are distributed at S5 and S6, which are close to soma and have moderate dendrite diameters, NMDAR inhibits the excitability and depresses somatic spikes. For the synapses located at S9 and S10, which are the closest to soma and have the largest dendritic diameter, the somatic voltage is bursting with NMDARs, and additional NMDARs make PC membrane potential saturated with too much excitation inputs.

With this initial screening of dendritic location, three sites (S4, S6, and S9) are selected to further investigate the effect of the number of NMDARs on each location (Fig 1C). With the same Poisson stimulus, the NMDAR number is varying from 50 to 800 synapses on each site (Fig 1D). PC somatic response to synaptic input without NMDAR (baseline) shows different profiles with increasing NMDAR numbers. Compared to the baseline, NMDARs show either more excitation or suppression on somatic spikes. NMDARs enhance simple spikes at S4, but suppress simple spikes at S6. For S9 closer to soma, NMDARs induce somatic complex spikes. Such results are observed with different dendritic morphologies of rat and mouse PCs (S1 Fig) and different stimulation protocols (S2 Fig). The diverse response induced by NMDA is also intertwined with intrinsic ion channel properties, particularly the A-type K^+^ channel (S3 Fig). These results indicate that dendritic NMDARs regulate somatic response, yet depending on the location of dendrites. As a result, a large diversity of somatic responses is displayed.

### 2.2 The effect of the random or clustered distribution of dendritic NMDARs

**Fig 2:**
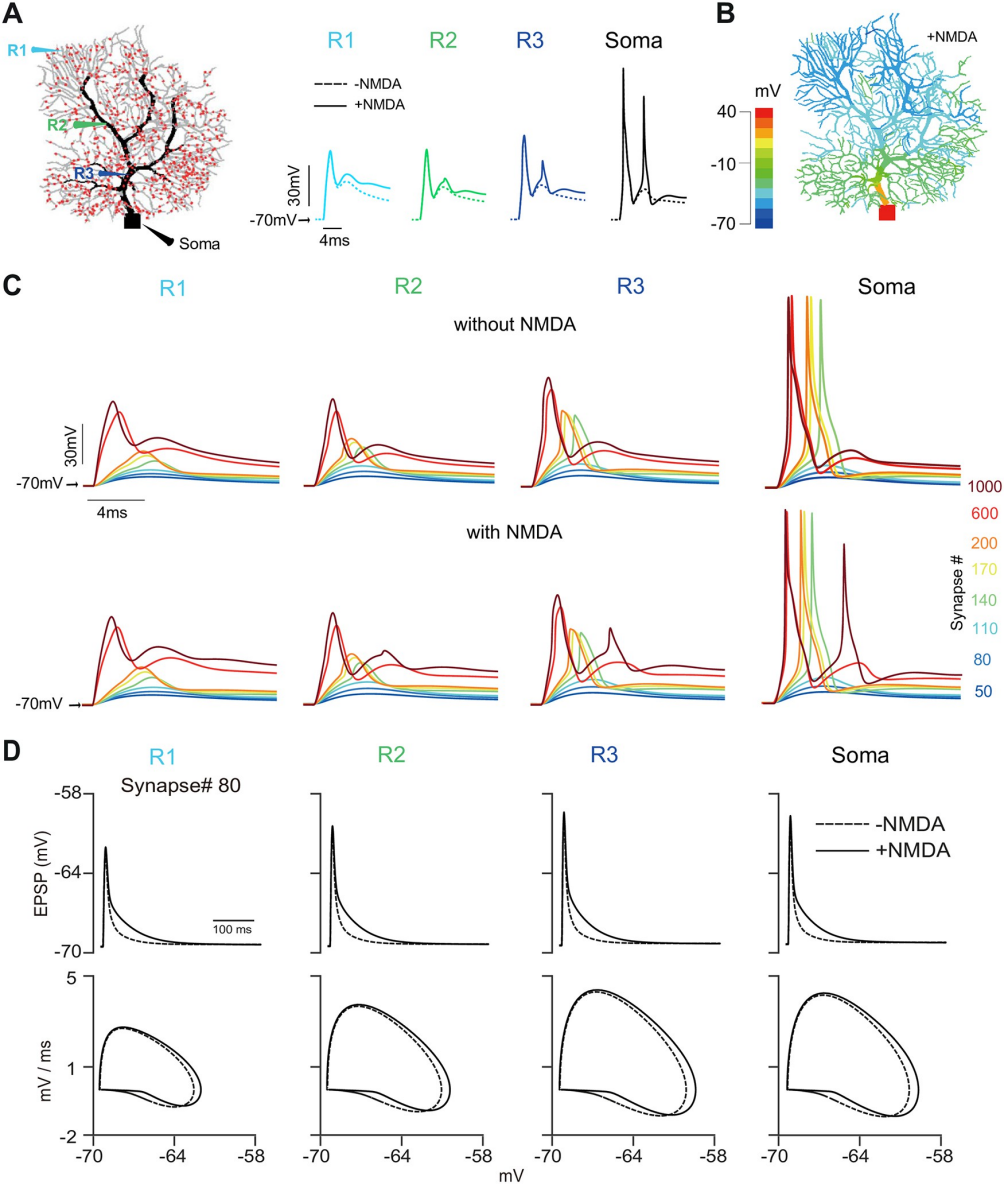
Dendritic and somatic responses under the randomly distributed NMDARs. (A) Response recorded at different locations. (Left) four recording sites: R1 (spiny), R2 (distal), R3 (proximal) dendrite, R4 (soma). 1000 synapses (red dots) randomly distributed on the PC. (Middle) Voltage responses recorded at four sites with and without NMDARs. (B) The distribution of peaks of voltage potential triggered in (A) with NMDARs installed. (C) Response profiles at four sites varied with the number of input synapses (50 to 1000) with and without NMDARs. (D) The profiles of EPSP and the phase plots of voltage change by NMDARs.

Dendritic computing has been studied intensively to see if synapses are more randomly or clustered distributed over dendrites. Here we consider this question using a similar protocol. First, when 1000 synapses are randomly distributed over the entire dendritic tree of a PC, membrane potentials at different dendritic sites (R1–R3) and the soma are recorded (Fig 2A). Three sites, R1 to R3, have a closer distance to soma. The peak of the membrane potential is inversely proportional to this distance. R3 dendritic spikes become larger compared to those at R1, which is more prominent when NMDARs are installed. NMDARs help to shape the later and prolonged response to incoming stimuli. As a result, MMDARs enable the soma to generate a secondary spikelet. At the time point of somatic spike timing, the snapshot of voltage values over the entire dendritic field shows that voltages are decreased gradually from soma to spiny dendrites (Fig 2B). Similar profiles, shorter spike latency, and higher voltage peaks, are still found when changing the number of synapses (Fig 2C). For the example of 80 synapses (Fig 2D), and more examples (S4 Fig), triggered dendritic and somatic responses (Excitatory postsynaptic potentials; EPSPs) are faster and larger because of NMDARs as shown by a phase plot. The synaptic integration window is significantly increased after adding NMDARs.

**Fig 3:**
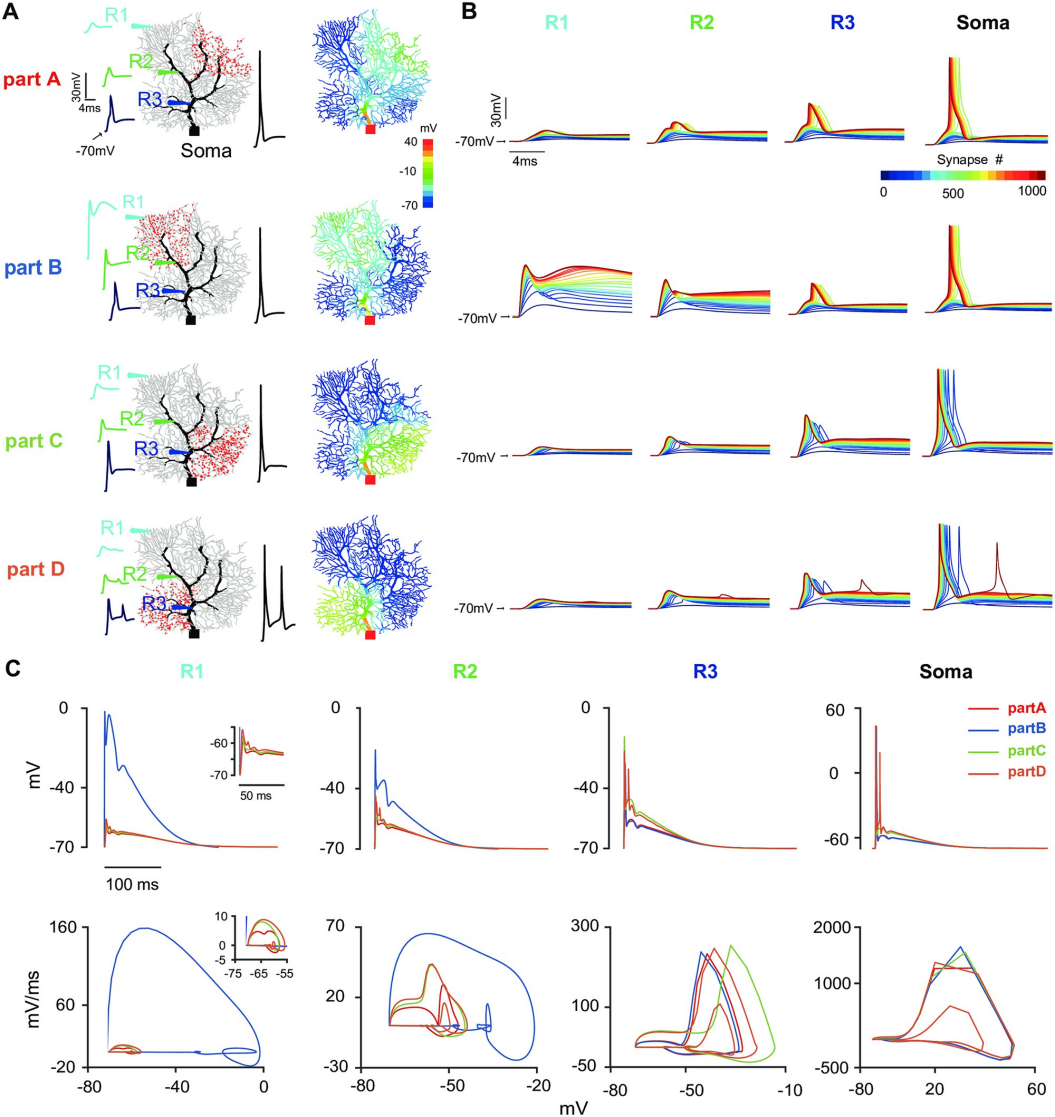
Regulating response at different parts of dendritic fields by NMDARs. (A) Regional synaptic distribution and dendrite potential profile. (Left) Synapses (1000 red spots) distributed in four regions (parts A-D) respectively. Insets are triggered voltage traces at each recording site (S1–S3 and Soma). (Right) The corresponding distribution of peaks of voltage potential. (B) Voltage profiles at four sites varied with the number of input synapses. (C) The corresponding phase plots of voltage profiles with 1000 synapses.

Compared to the random distribution, our above results suggest that specific locations of afferent synapses influence the somatic response. Now we consider a more clustered distribution of synapses with NMDARs included. PC, as a nice model cell, shows complex dendritic branching. There are roughly four regions (parts A-D), where parts A and B are far from the soma, whereas parts C and D are close to the soma (Fig 3A). After distributing 1000 synapses over each part, the dendritic and somatic responses are different. Similarly, dendritic voltage responses decrease with the distance from the soma (Fig 3A, voltage traces on the left). The distribution of voltage values at the time of somatic spikes over the entire dendritic fields shows the prorogation of stimuli towards soma (Fig 3A, right).

Similar response properties are observed when the stimulus intensity, the number of synapses, changes in Fig 3B, except for part B where R1 and R2 are located. Compared between different parts, somatic responses are remarkably different. The synaptic activation causes a stronger depolarization in parts C and D than in parts A and B. Accordingly, different numbers of synapses are required to trigger somatic spikes. The distance to soma defines the minimum number of synapses required to generate a somatic spike. Thus, it is reasonable that the synaptic location could dynamically adjust the somatic firing threshold. Secondary dendritic spikelets appear in part D only. The corresponding phase plots of voltage profiles at different recording sites and stimulation parts show the detailed timing dynamics (Fig 3C). These results suggest that PC somatic responses depend on how synapses are distributed and stimulated.

### 2.3 Complex spikes induced by NMDAR

**Fig 4:**
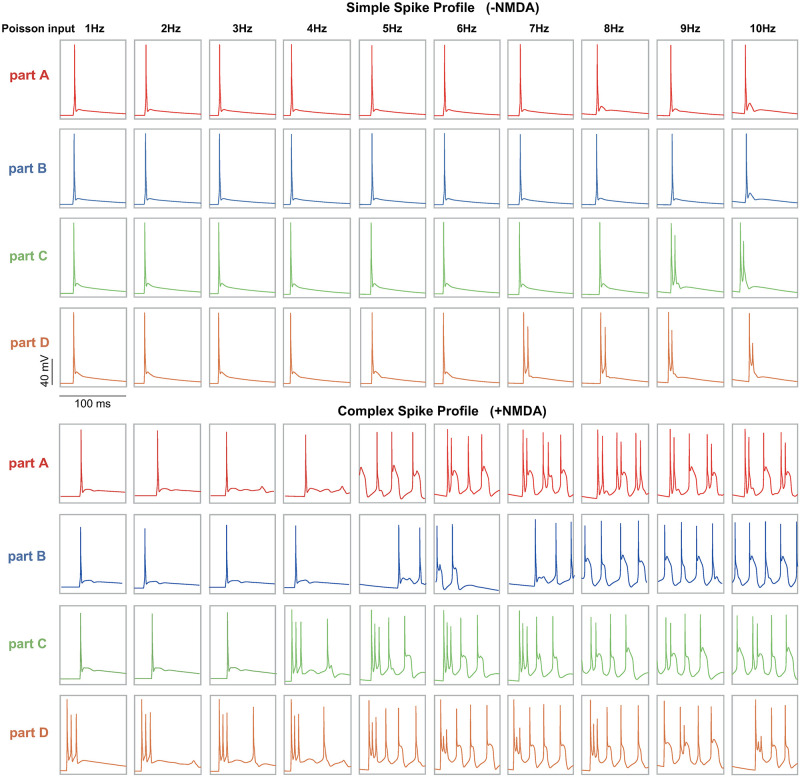
PC somatic response profiles induced by low-frequency Poisson inputs at different parts of dendrites. Simplex spikes are triggered in all parts, while complex spikes are shown in Part A, C, and D.

Above we demonstrated that there are secondary somatic spikes when NMDARs are distributed in part D. Recall both parts C and D are close to the soma. These dendritic parts have been thought of as the original source of complex spikes where climbing fibers come into the PC dendrites [37]. Compared to the high frequency of PFs, CFs usually exhibit a low-frequency input to PC. Indeed, we found that with a range of low frequencies of Poisson synaptic inputs, part D is easier to fire complex spikes (Fig 4). Such a profile can not be displayed when there is no NMDA.

In general, the occurrence of CS is related to the location of the synaptic input distribution, which is related to the formation of synaptic connection between CF and PC and the induction of CS ([38, 39]). In addition, CS features are stimulated by frequency modulation. Our example of the low-frequency stimulation in Fig 4 shows that increasing the stimulation frequency, CS can also appear in part C, then part A, whereas the stimulation part B did not induce any complex spikes at any frequency tested. In part A, a higher frequency is required to produce CS compare with parts C and D. Especially in part D, 1 HZ stimulation can induce a typical CS response. Traditionally, CF inputs induce CS response, and CF synapses are distributed in main dendrites, here part C and part D. Our results suggest that CS response can be evoked on spiny dendrites, which are consistent with recent experimental findings [40, 41]. Together with recent discussions on the location of NMDARs and interactions between simple and complex spikes, our results suggest the complex role of NMDARs on PC responses.

### 2.4 The effect of dendritic distance and diameter

**Fig 5:**
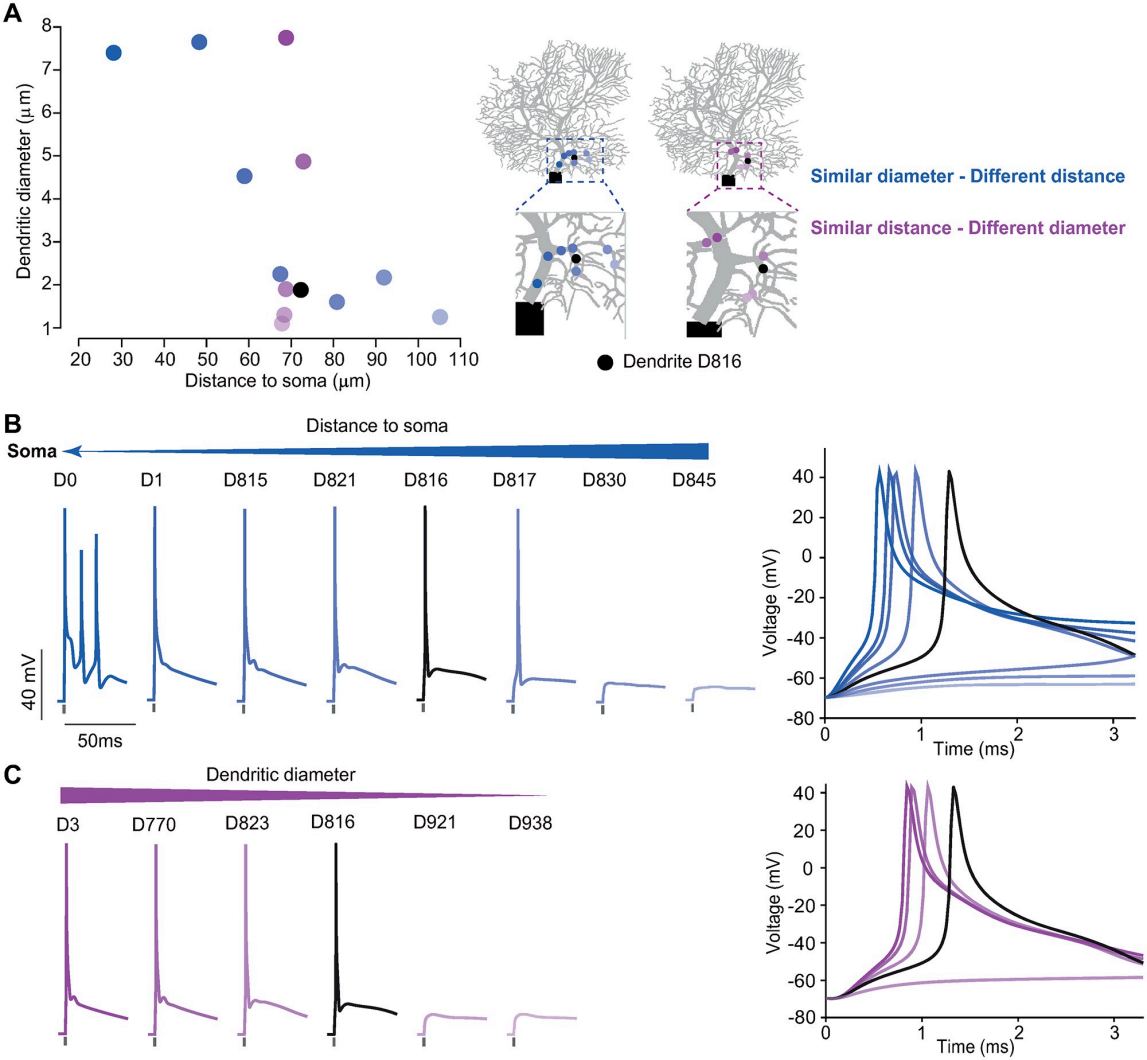
Somatic responses depending on the dendritic properties of diameter and distance to soma. (A) (Left) Different dendrites selected in part D that have either similar diameters (blue) or distance to the soma (purple) referring to the dendrite D816. (Right) Illustrating the locations of each dendrite: eight dendrites with different distances to soma; six dendrites with different diameters. (B) PC somatic response decreased with the increasing distance of the dendrites stimulated. (Right) The inset of enlarged membrane potential responses starting at the time of onset stimulation. For illustration, voltages were cut off at 40 mV. (C) Similar to (B), but for the case of varying dendritic diameters.

Next, we conduct a more detailed analysis by controlling synapses distributed on individual dendrites with different diameters and distances from the soma (Fig 5). Firstly, we select 8 dendrites where the distance increases sequentially, accompanied by a decrease in the diameter of dendrites (Fig 5A, S1 Table). Increased distance results in a delayed somatic response (Fig 5B). In particular, the distribution of synapses in the proximal and larger diameter dendrites (D0) evoked CS production. However, synaptic inputs can not induce spikes when they are distributed on distal dendrites (D830 and D845). Smaller diameter dendrites (D816 and D817) compared with D830 also generate spikes. In contrast, we also select another group of 6 dendrites, showing similar distances, but with decreasing diameters(Fig 5A, S2 Table). We also found the delayed response with decreasing diameter (Fig 5C), where synaptic inputs fail to induce spikes in small-diameter dendrites. Narrow diameters also produce large local input resistances, which are responsible for large local synaptic depolarizations. This depolarization reduces the driving force for synaptic current. Our results, consistent with classical studies on dendritic computing, indicate that the overall balance between the distance and diameter of dendrites is critical for somatic responses.

### 2.5 Combined PF and CF synapses

**Fig 6:**
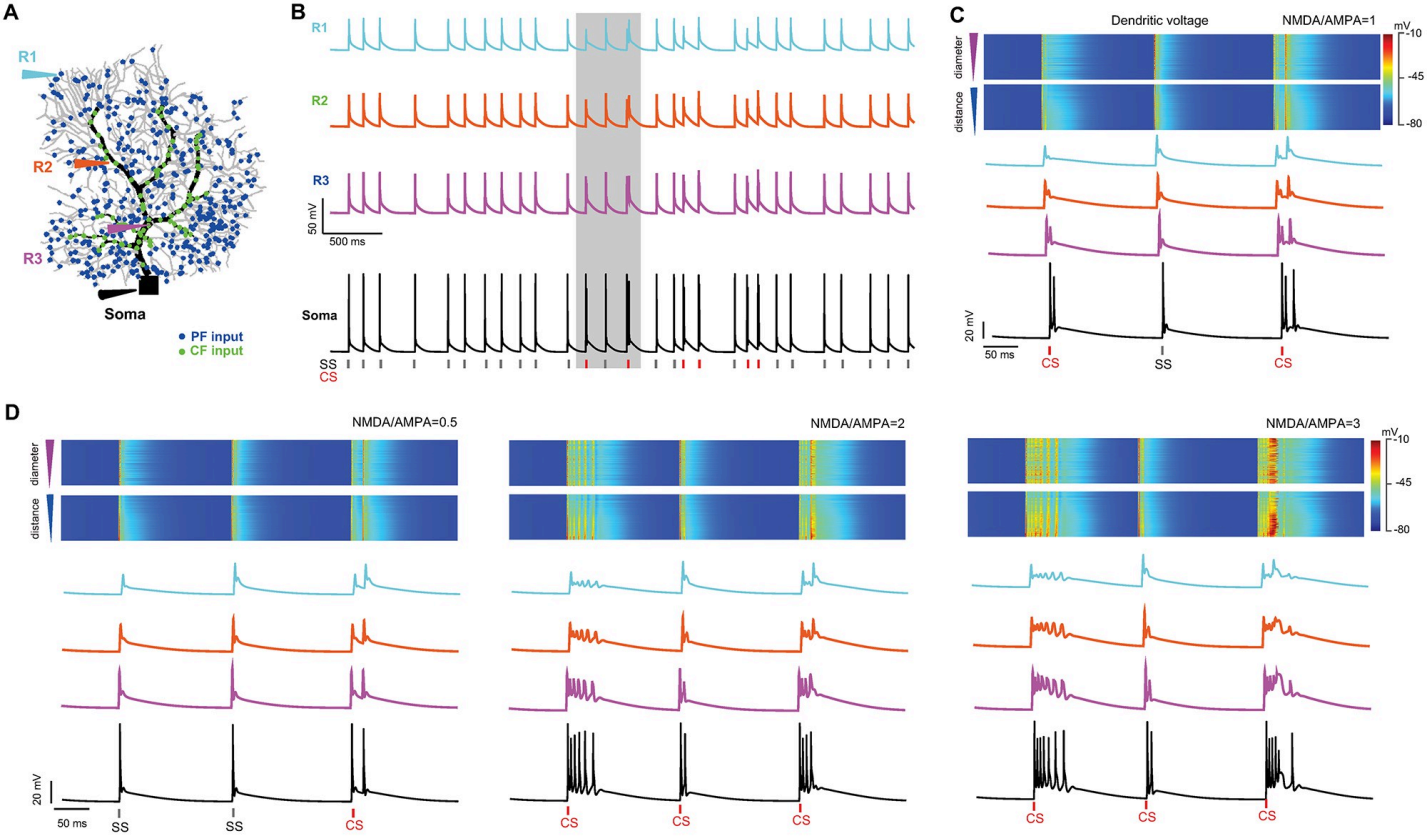
PC response to combined PF and CF inputs. (A) PC installed with 500 PF AMPA synapses stimulated at 5 Hz Poisson spikes and a CF with 500 AMPA+NMDA synaptic connects stimulated at 2 Hz. Four recording sites (R1, spiny; R2, smooth; R3, trunk dendrites; Soma). (B) Voltage response of dendrites (R1–R3) and the soma. Spike timings of SS (black) and CS (red) are indicated as ticks. (C) Voltage response profiles zoomed in at a period (gray box in (B)) from all dendrites, sorted by their diameters and distances to the soma (top), and (below) profiles of each of four sites (R1–R3 and soma). (D) Similar to C, but with the changing strength of NMDA at different ratios of NMDA/AMPA while AMPA is fixed.

Next, we consider a scenario where both PF and CF synapses are stimulated in a PC (Fig 6). 500 PFs were randomly distributed on spiny dendrites and each was modeled by AMPA synaptic dynamics and independently stimulated with 5 HZ Poisson spikes. A single CF was with synaptic contacts at smooth and trunk dendrites, and each was modeled with both AMPA and NMDA dynamics and simultaneously stimulated at a low frequency of 2 Hz Poisson spikes [37, 42]. With such a mixture of input protocol (Fig 6 A), membrane voltage responses at the soma and three different dendritic sites were recorded. Similar to experimental observations of spontaneously active PCs in awake mice [40], a sequence of spikes with both SS and CS is generated at dendrites and soma (Fig 6B and 6C). When changing the strength of NMDA by varying the ratio of NMDA/AMPA, the amplitude of complex spikes was changed proportionally. As a result, NMDA contributes to complex spikes significantly (Fig 6D).

**Fig 7:**
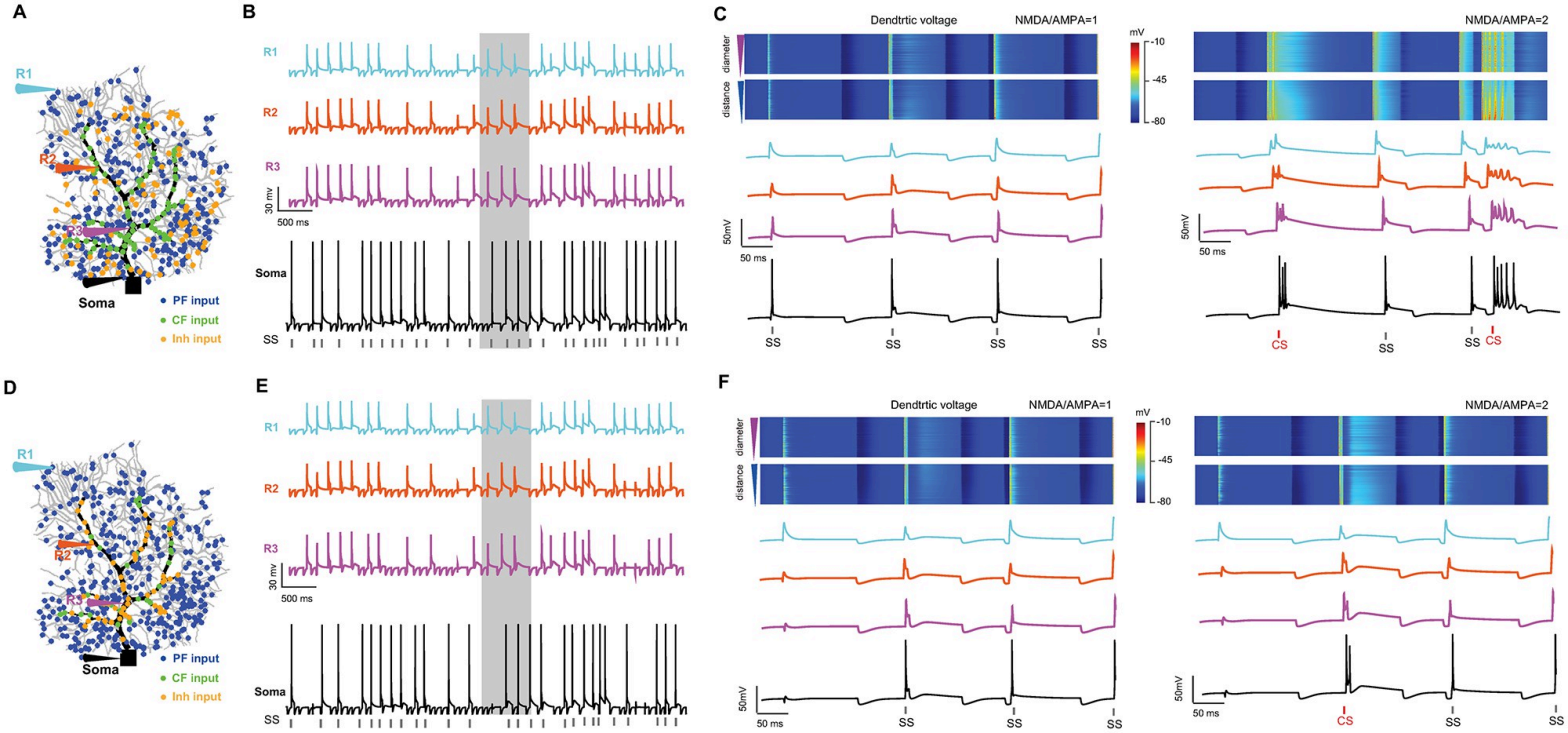
PC response to combined PF and CF inputs with inhibition input. (A-C) Similar to Fig 6, but with 200 inhibitory synapses distributed on spiny dendrites at 10 Hz Poisson spike stimulation. (D-F) Similar to (A-C) while inhibitory synapses are distributed on smooth dendrites.

We then added additional inhibitory synapses distributed over spiny dendrites close to PFs (Fig 7A). Together with excitation, inhibition can regulate the discharge profile of simple and complex spikes (Fig 7B and 7C). When inhibitory synapses are distributed on smooth dendrites close to the CF, PC firing patterns are regulated differently, in particular when the NMDA strength is stronger (Fig 7D, 7E and 7F). Such observations are more potent when the resurgent Na^+^ channels are included in the PC model to make the PC generate spontaneous firing (S5 Fig). These results are consistent with the experimental observation of the role of inhibition in regulating PC firing [43].

## 3 Discussion

At Purkinje cells, NMDA receptors are considered abundantly existing in CF-PC synapses [16]. Recent evidence suggests they play an important role in PF-PC synapses [16, 18, 22–25]. Studying the role of NMDARs in neural dendritic and somatic responses is beneficial to enrich the information transmission mechanism between neurons and provide a reference for studying synaptic integration ability in single neurons. In this study, we utilized the PC as a model system to investigate the diverse role of NMDARs in dendritic integration.

### 3.1 The role of NMDAR is affected by the characteristics of dendrites

As the core of neural circuit function, nonlinear integration and saturation of dendrite mechanisms enhance the ability of single neurons to adjust synaptic input [44]. Individual dendritic branches can either linearly integrate inputs or generate localized all-or-none dendritic spikes depending on the modifiable branch excitability [31]. Thus, the distribution of NMDARs may be the decisive factor affecting the information transmission of neurons [28]. Consistent with previous studies, we show that cell morphology makes the dendrites show uneven excitability in a single branch [30, 45]. It is largely due to that the reliable propagation of the spikelet to the cerebellar nucleus can be guaranteed only when the dv/dt peak of the first spikelet of the dendrites is large [46, 47]. We distributed NMDARs to different regions of dendrites. Depending on the different characteristics of dendrites, our results show that the larger the diameter of dendrites and the closer to soma, the stronger the inhibition of the NMDAR effect. Our results further clarify the detailed dendritic regulatory relationship using a more complex PC model, illuminate the specific response of PC to input, and the forms of PC activity-dependent synaptic and non-synaptic plasticity selectively regulating dendritic input processing at the level of dendritic subdomains [48].

In this study, we use Purkinje cells to demonstrate the role of NMDARs on dendritic integration. From the structure view, PCs have a very large and rich dendritic structure. From the functional view, PCs have well-defined simple and complex spikes that have been shown depending on inputs at dendritic locations. Compared to PCs, other cells in the cortex that have been studied for dendritic integration have relatively small and simple dendritic structures. Although neuronal morphology can be dramatically different across cells in the cerebellum and cortex, the underlying dendritic properties are similar (PC vs. cortical L3, L4, and L5 cells in S6 Fig). For cortical cells, NMDARs have been thought as an important role in nonlinear dendritic integration [49, 50] and have some specific functions for input [51–54]. It has been found that spiny dendrites could possess NMDAR-mediated supralinearity [50, 55, 56]. However, recent evidence suggests that this superlinearity could depend on specific dendrites in the same L5 pyramidal neurons of the retrosplenial cortex [57], where basal and tuft dendrites exhibit superlinearity while apical oblique dendrites show linear integration. As for cerebellar neurons, experimental evidence indicates that stellate cells exhibit sublinear integration [58, 59]. Our results are consistent with these recent experimental results supporting diverse modes of NMDAR for distinct dendritic integration. Yet, it remains to be examined the functionality of the nonlinearity profile of Purkinje cells depending on the dendrites [59].

### 3.2 Interaction between different synaptic inputs

For neuronal somatic spiking responses, there are two fundamental firing modes as regular and bursting spikes [60–62]. In the case of PCs, regular or simple spikes are mostly triggered by parallel fibers, while bursting or complex spikes are induced by climbing fibers [63–66]. A single CF makes approximately 500 synaptic contacts with PC proximal dendrites, discarding at a low frequency [37, 42]. However, the interaction between these two types of fibers is more complicated than expected. The combined activity of PF and CF synaptic input, together with intrinsic dendritic mechanisms, allow for a full dynamic spatiotemporal range in neural responses [41].

It has been found that the spiny PC dendrites have rich local signaling resulting from clustered PF input, which can further be summed to make local regenerative events [40]. However, the mechanism behind this is not clear, our results suggest NMDARs, together with clustered PF inputs, could contribute to the complex spikes response. Together with the heterogeneous CF activity, both PF and CF could be intervened for PC activity.

Distal dendrites, far end form soma, are unlikely to be innervated by CFs. Their responses shown in this work are mainly the products of dendritic filtering. Recent studies show that PC spiny distal dendrites exhibit CS responses [40]. Consisting with these experimental observations, we also found that distal dendrites can display CS as long as NMDARs are expressed. Even more, different locations, from distal to middle and proximal dendrites, all exhibit the CS response in the resting state of awake mice [40] and natural sensory stimulation [41].

Furthermore, inhibition input also plays an important role in regulating neuronal response [43, 67, 68]. The interaction between inhibition and excitation could be excited in a complicated way depending on the inhibitory input locations [67–69]. For Purkinje cells, the major inhibition comes from stellate cells and basket cells, in which stellate cells tend to target PC distal dendrites, whereas, basket cells mainly target deeper parts of PC somas [70]. Recent experiments suggest that basket cells are more potent than stellate cells in inhibiting PC spiking while both types of cells deliver a continuum of inhibition across the entire depth of the molecular layer [43]. Although the explicit modeling of stellate cells and basket cells was not conducted in our current work, our results are consistent with the picture that inhibition at different parts of PC regulates PC firing in a different way, particularly when excitation of NMDA is stronger (Fig 7).

### 3.3 Limitations

As mediators of information transmission between neurons, NMDARs play a key role in synaptic information transmission in neural circuits [4, 5, 8]. Our current work only considers the coding properties of individual neural response computing units regulated by NMDARs. As a key element of the connection components between neurons in neural networks, studying the effect of NMDARs on the information transmission in the neural network will be more conducive to improving the role of NMDARs in the integration of cerebellar information. Due to typical cellular morphology, neurons show branch-specific responses to different intensities of input. Dendritic plasticity can be triggered by patterns of synaptic or non-synaptic activity and may be limited to the active regions of dendritic trees [48]. Therefore, it is of great significance to explore the effect of NMDARs on dendritic plasticity [54, 71, 72].

Besides the plasticity of circuit mechanisms, the diverse role of NMDARs for dendritic integration is also regulated by voltage-dependent ion channels. Such regulation could reshape synaptic integration profiles and rectify the nonlinear integration to be more linear [73, 74]. Together with the diverse linear and/or nonlinear synaptic integration at different parts of dendrites in the cerebellar and cortical cells [57–59], future work is deserved to explore diverse modes of dendritic integration with a functional implication for neuronal computation.

## 4 Methods

### 4.1 Neuronal model

To investigate the mechanisms and effects of NMDA synapse on the PC responses, and the relationship between PC morphology and NMDA receptors expression, a detailed compartmental model of a cerebellar PC with active dendrites was constructed. We used a 3D reconstruction of a guinea-pig PC available on the public archive www.neuromorpho.org (NMO-00610). To ensure a representative range of morphological properties, we also examined two additional PCs from rat (NMO-00891) and mouse (NMO-00865), with different morphologies. Each neuron contains two major parts: dendrites and soma. The division of dendrites is based on the Strahler strategy [75].

The PC was modeled with the same parameters as before [75]. Briefly, the model has the following passive parameters: membrane resistance *R*_*m*_ = 5000Ω/*cm*^2^, axial resistance *R*_*i*_ = 250Ω/*cm*. membrane capacitance *C*_*m*_ was set to 0.8uF/*cm*^2^ in the soma, trunk dendrites, and smooth dendrites, and 1.5uF/*cm*^2^ in spiny dendrites. There are 13 different types of voltage-gated ion channels modeled, eight of which (P-type *Ca*^2+^ channel, T-type *Ca*^2+^ channel, class-E *Ca*^2+^ channel, persistent *K*^+^ channel, A-type *K*^+^ channel, D-type *K*^+^ channel, delayed rectifier, decay of sub-membrane *Ca*^2+^) were inserted into the soma and dendrites. In addition, three ion channels (fast *Na*^+^ channel, persistent *Na*^+^ channel, anomalous rectifier channel) were solely added to the soma, and two ion channels (high-threshold *Ca*^2+^-activated *K*^+^channel, low-threshold *Ca*^2+^-activated *K*^+^ channel) were solely added to the dendrites. These channels used standard Hodgkin-Huxley formulation. The resting potential of the neuron was set at -65mV and the temperature was set at 37°C. For S5 Fig, the additional resurgent *Na*^+^ channel was applied to both soma and dendrites with the model parameters from [76].

To demonstrate the general dendritic profile of different cell types, we modeled three additional cortical cells as in S6 Fig: L5 pyramidal cell, L4 spiny stellate cell, and L3 pyramidal cell taken from [77]. These cortical cells have much fewer dendrites than PC while exhibiting similar biophysical properties.

### 4.2 Synapse model

In our model, we randomly distributed 1000 synapses on the spiny dendrites to receive stimulus input from PF, including three types of receptor models, namely AMPA (I), NMDA (II), and AMPA+NMDA (III). And in the third receptor model, AMPA and NMDA channels were colocalized one-to-one at each synapse and received the same inputs [78]. AMPA and NMDA receptors are two excitatory receptors, which can mediate excitatory postsynaptic currents and have complex dynamics. Two state kinetic scheme synapse described by rise time constant *τ*_*rise*_, and decay time constant *τ*_*decay*_. Accordingly, the mathematical model of AMPA/NMDA receptor is *I*_*r*_ = *g*_*r*_ × (*V* − *E*_*r*_) with *g*_*r*_ = *g*_max_*r*_ × *weight* × *factor* × *Q*_*r*_, where, *r* ∈ {AMPA, NMDA}, *I*_*r*_ is the receptor current, *g*_*r*_ is the receptor conductance, *V* is the synaptic membrane potential, *E*_*r*_ is the receptor reversal potential, *g*_max_*r*_ is the maximum synaptic conductance, *weight* is the connection weight between the synaptic stimulus and the neuron, *factor* is to make the normalized peak of the conductance 1. Since NMDA-mediated currents often require AMPA-mediated depolarization to remove extracellular *Mg*^2+^ blockade of NMDA-associated channels [79], the associated channels open only when magnesium ions are blocked and NMDA receptors are activated. Therefore, the configuration parameter Q_*r*_ of different types of receptor models is

$$\begin{array}{c} Q_{AMPA}=e^{-\left(t-t^{f}\right)/\tau_{decay}}-e^{-\left(t-t^{f}\right)/\tau_{rise}} \end{array}$$

$$\begin{array}{c} Q_{NMDA}=\left[e^{-\left(t-t^{f}\right)/\tau_{decay}}-e^{-\left(t-t^{f}\right)/\tau_{rise}}\right]\times g_{Mg^{2+}} \end{array}$$

where, *t*^*f*^ is the moment when the stimulus arrives, *g*_*Mg*^2+^_ represents the channel controlled by *Mg*^2+^. Both AMPA and NMDA receptors were double exponential models, and NMDA receptor model was more complex and had more dynamic behaviors than AMPA receptor model.

For the inhibitory synaptic input, a model of GABA_A_ synapse was applied with similar dynamics as the AMPA synapse, except that the parameters were adjusted to be inhibitory as in [65]. Detailed model parameters of AMPA, NMDA, and GABA_A_ receptors are listed in Table 1.

**Table 1:** Parameters for synaptic currents.

Synape	g(ns)	E(mV)	*τ*_*rise*_ (ms)	*τ*_*decay*_ (ms)
AMPA	0.8	0	0.5	5
NMDA	0.96	0	8	30
GABA_A_	2	-85	1.8	8.5

### 4.3 Stimulus protocol

Synaptic inputs were modeled using a modified version of the *NetStim* object provided in the NEURON package. Each synapse received an independent spike train generated simultaneously [78]. A single stimulation consists of a sequence of spikes containing spike times and inter-spike intervals, so we can generate a successive spike train by the previous spike plus the regular or irregular time intervals. Each spike train was generated using the same algorithm as in [75].

### 4.4 Data analysis

All the simulations were conducted with NEURON 7.6. The model output was analyzed with MATLAB 2017. The time step of all simulation experiments was set at 0.025ms. A simple spike is considered to occur when the membrane potential at its location crosses a threshold voltage (-10mV) in the positive direction, and the moment of the intersection is the timing point when a simple spike is generated. Similarly, a complex spike can be defined by the first timing point where the threshold is crossed.

## Supporting information

S1 Fig Related to Fig 1. Diverse somatic responses depending on the dendritic location of NMDARs in the additional rat (top) and mouse (bottom) PCs with different neuronal morphology.
(TIF)

S2 Fig Related to Fig 1. Diverse somatic responses with different frequencies of burst inputs.
(TIF)

S3 Fig Related to Fig 1. Diverse somatic responses with and without the A-type *K*^+^ ion current, which contributes to suppressing spiking together with NMDAR.
(TIF)

S4 Fig Related to Fig 2. The profiles of EPSP and the phase plots of voltage change by NMDARs at four sites with different input synapses.
(TIF)

S5 Fig PC firing patterns with spontaneous activity induced by resurgent Na^+^ ion channels. (A) Related to Fig 1. Diverse somatic responses depending on the dendritic location of NMDARs. (B) Related to Figs 6 and 7. Discharge of simple and complex spikes regulated by excitation and inhibition. NMDA/AMPA = 1.
(TIF)

S6 Fig Related to Fig 5. Similar properties of dendrites in different types of neurons. (A) Dendritic diameter vs. dendritic distance to soma. Each data point in the scatter plot represents a single dendrite in the NEURON model. Compared to the Purkinje cell (943 dendrites), three cortical cells have few dendrites (83, 80, and 104 dendrites). (B) The peak amplitude of EPSP (excitatory postsynaptic potential) induced on every single dendrite decays with the distance to soma. (C) The distribution of EPSP peak amplitude over the entire dendritic field. For the scaling bar, max peak values (0.5 mV for PC, 60 mV for L5, 50 mV for L4 and L3) are varying and are colored according to each cell.
(TIF)

S1 Table Eight stimulus sites with different distances from soma but similar diameters. (XLSX)

S2 Table Six stimulus sites with different dendrite diameters but similar distances to soma. (XLSX)
