# Supplementary figures and images for: Diverse role of NMDA receptors for dendritic integration of neural dynamics
Source: PLoS Comput Biol. 2023 Apr 10;19(4):e1011019. doi: 10.1371/journal.pcbi.1011019 (PMC10085026; doi:10.1371/journal.pcbi.1011019)

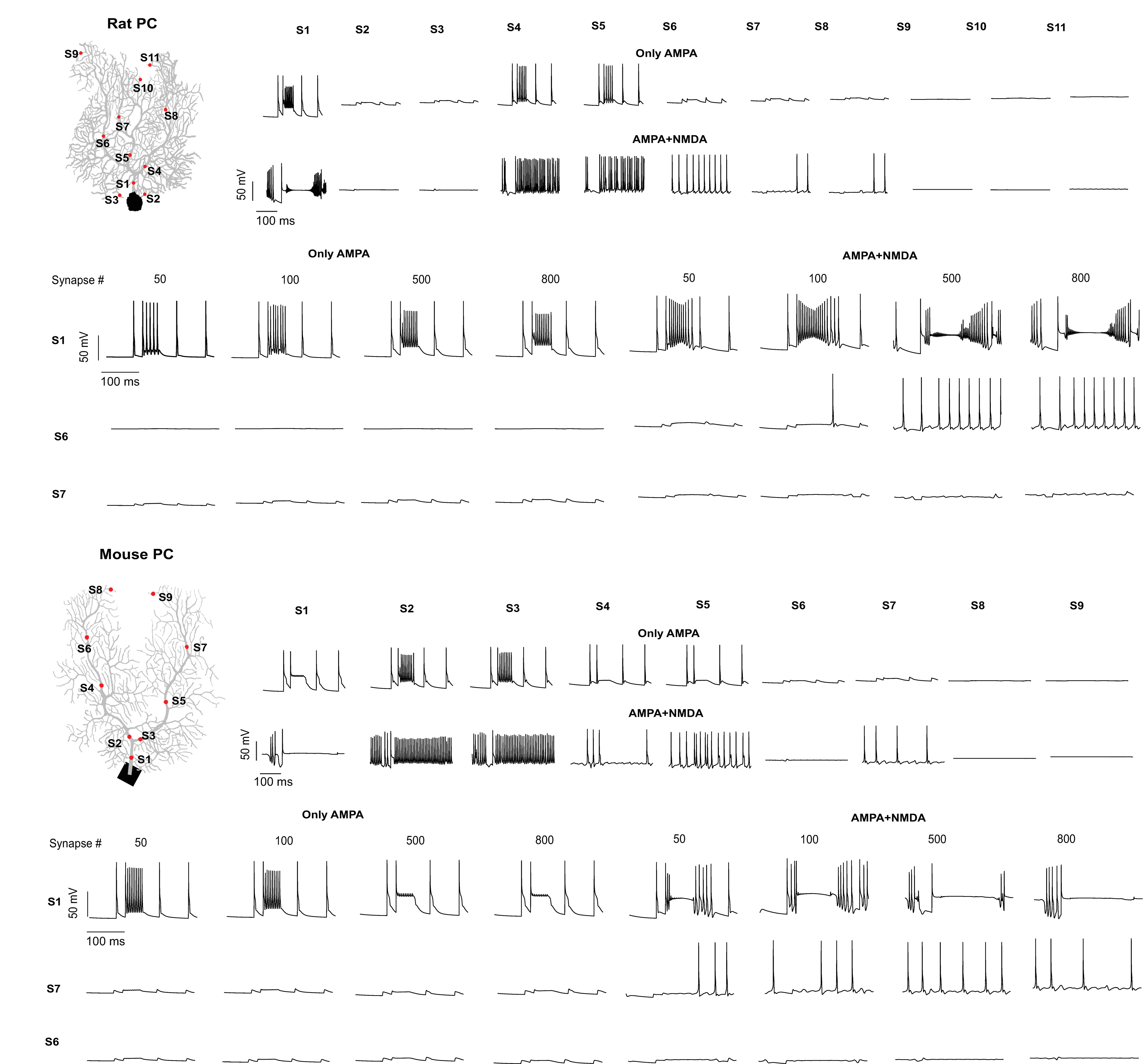

Supplement: S1 Fig — Diverse somatic responses depending on the dendritic location of NMDARs in the additional rat (top) and mouse (bottom) PCs with different neuronal morphology. (TIF) [file pcbi.1011019.s001.tif]

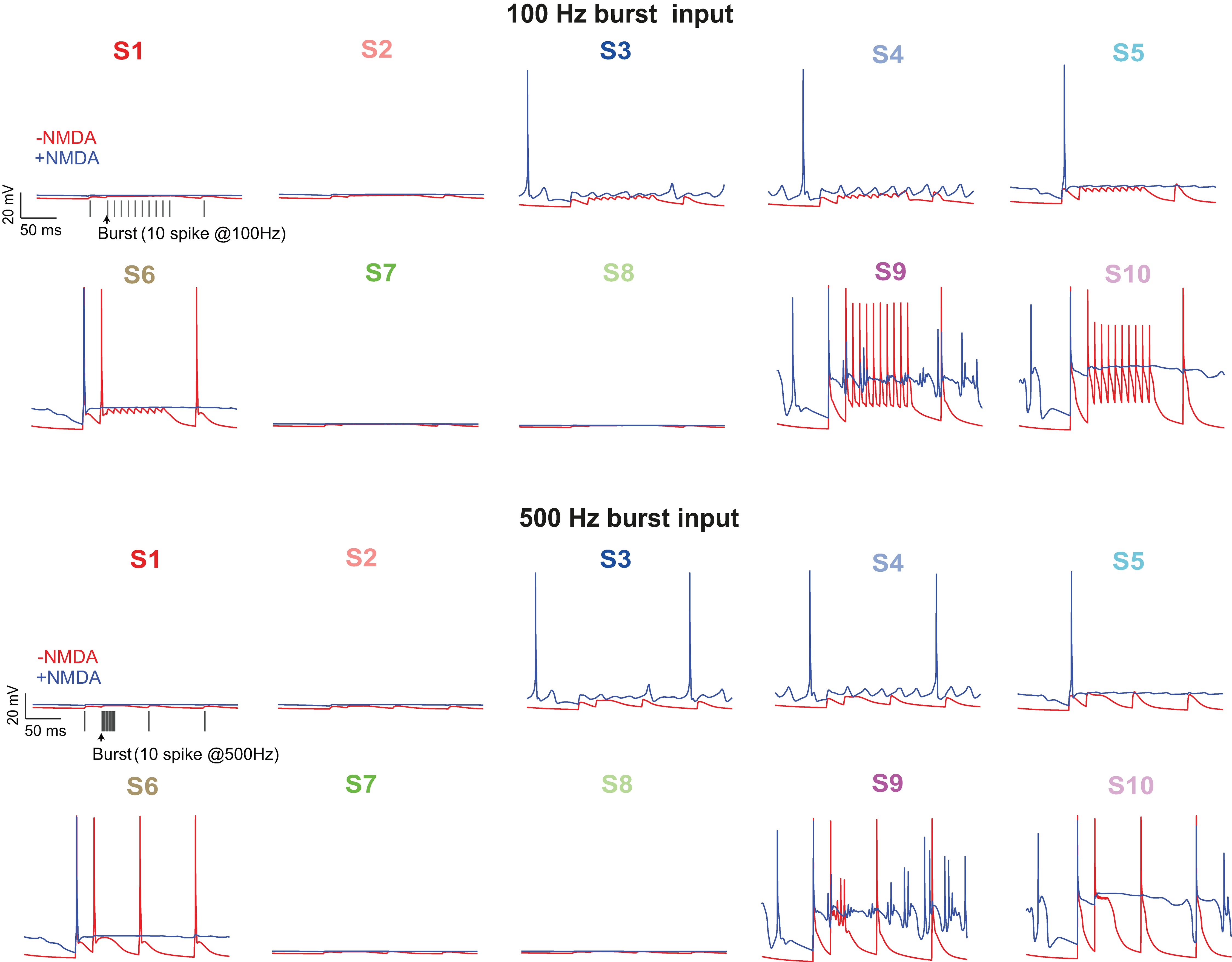

Supplement: S2 Fig — Diverse somatic responses with different frequencies of burst inputs. (TIF) [file pcbi.1011019.s002.tif]

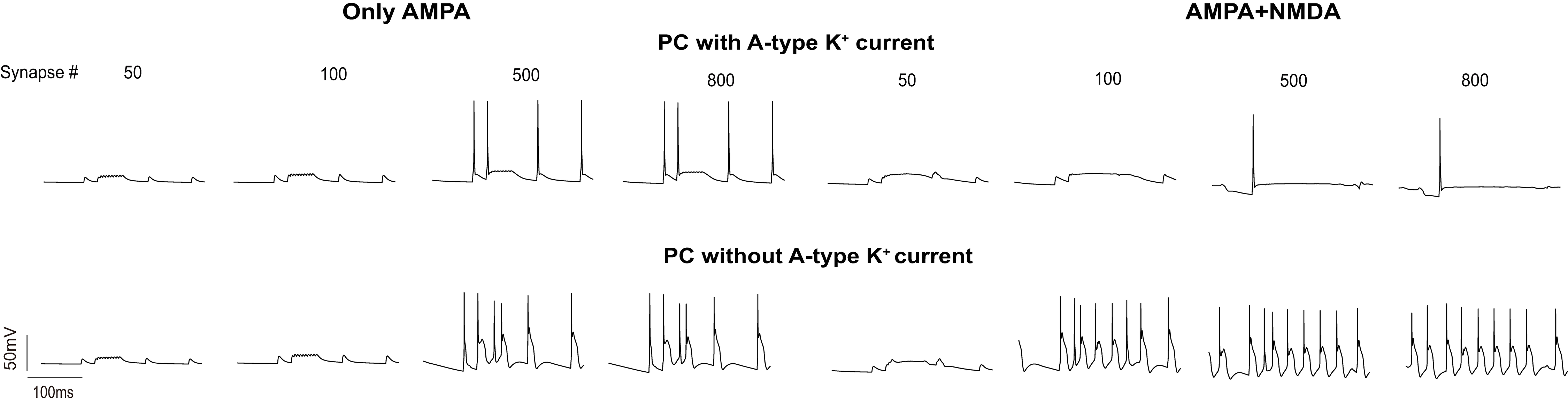

Supplement: S3 Fig — Diverse somatic responses with and without the A-type K+ ion current, which contributes to suppressing spiking together with NMDAR. (TIF) [file pcbi.1011019.s003.tif]

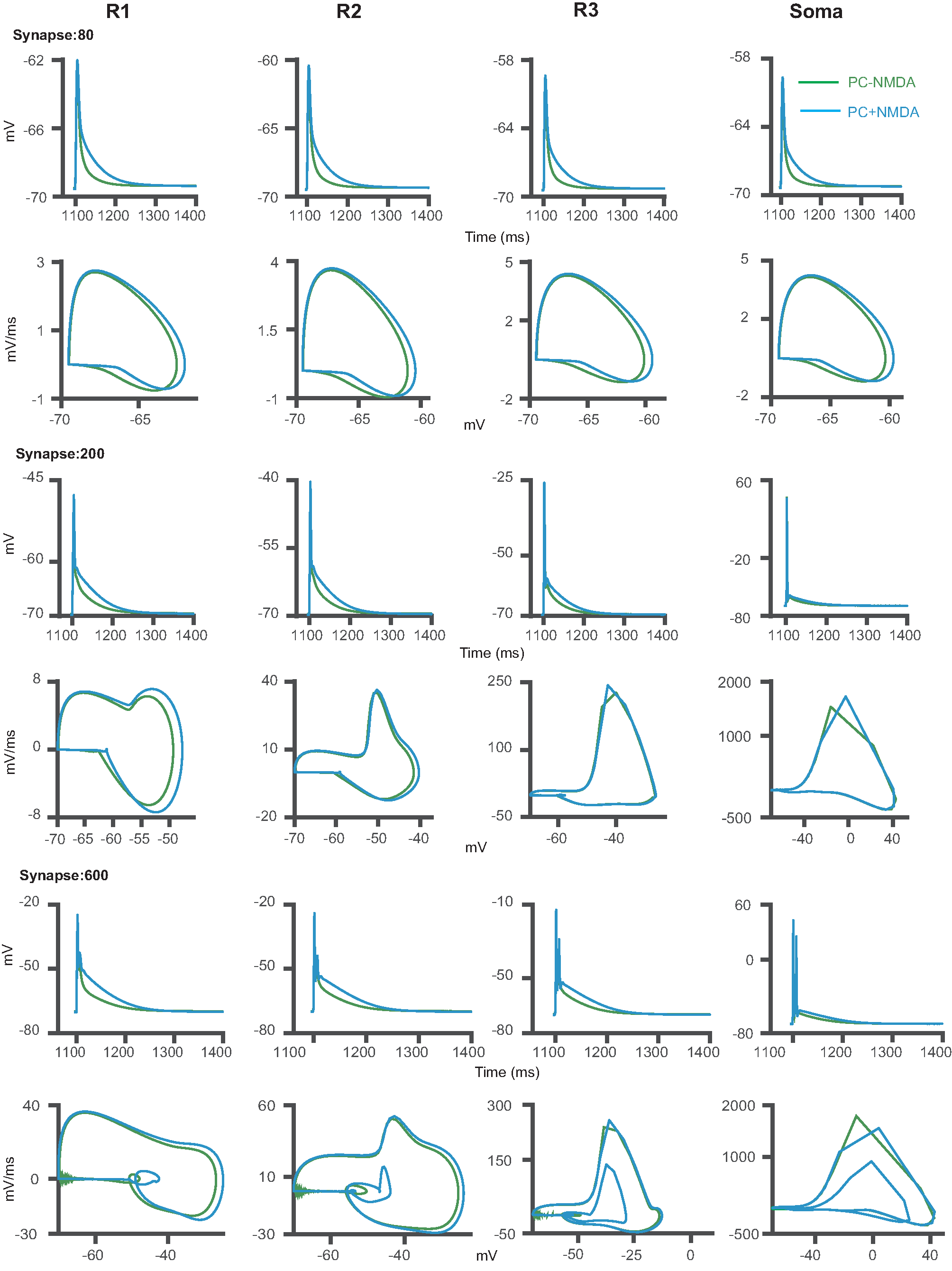

Supplement: S4 Fig — The profiles of EPSP and the phase plots of voltage change by NMDARs at four sites with different input synapses. (TIF) [file pcbi.1011019.s004.tif]

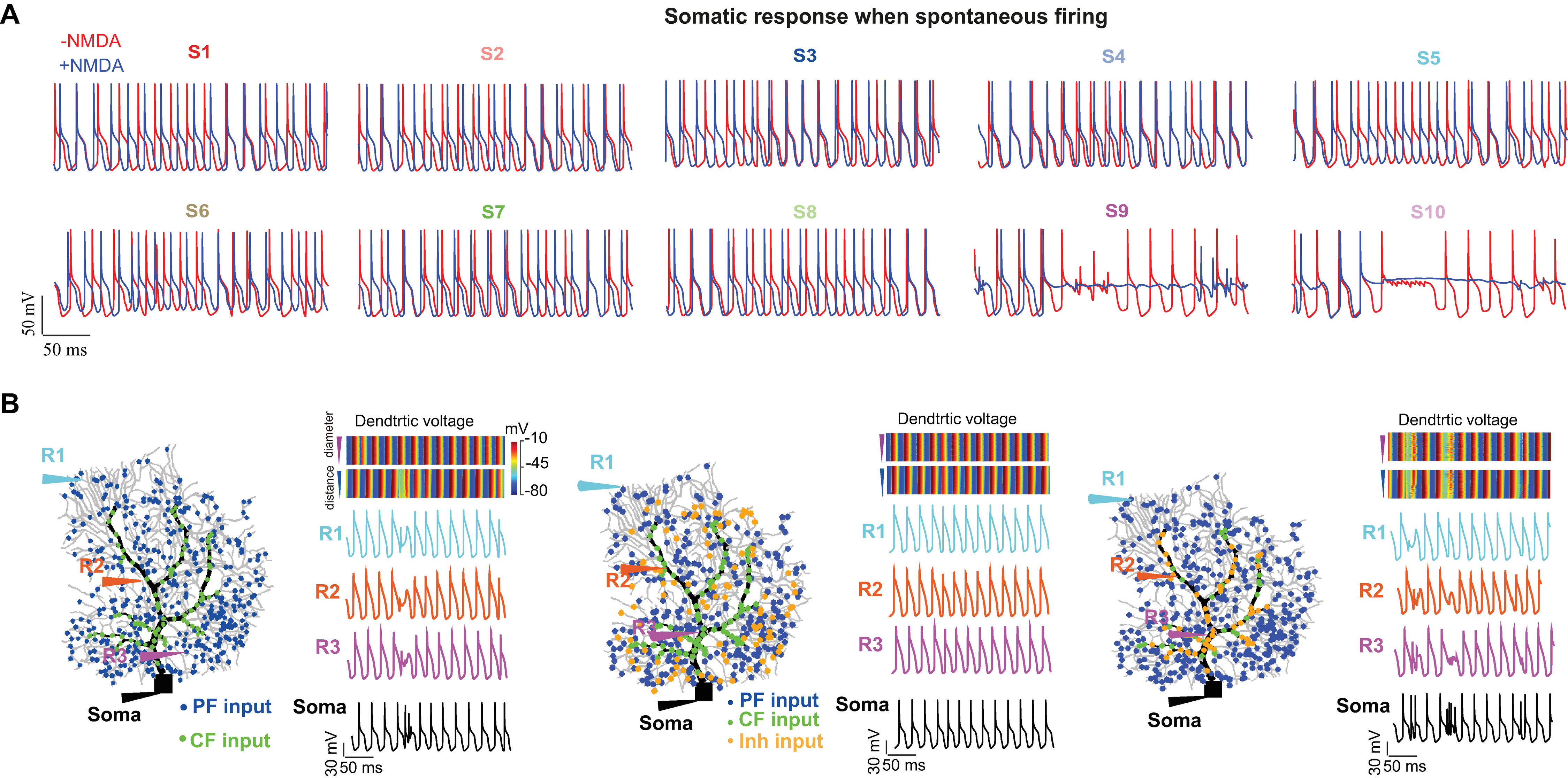

Supplement: S5 Fig — (A) Related to Fig 1. Diverse somatic responses depending on the dendritic location of NMDARs. (B) Related to Figs 6 and 7. Discharge of simple and complex spikes regulated by excitation and inhibition. NMDA/AMPA = 1. (TIF) [file pcbi.1011019.s005.tif]

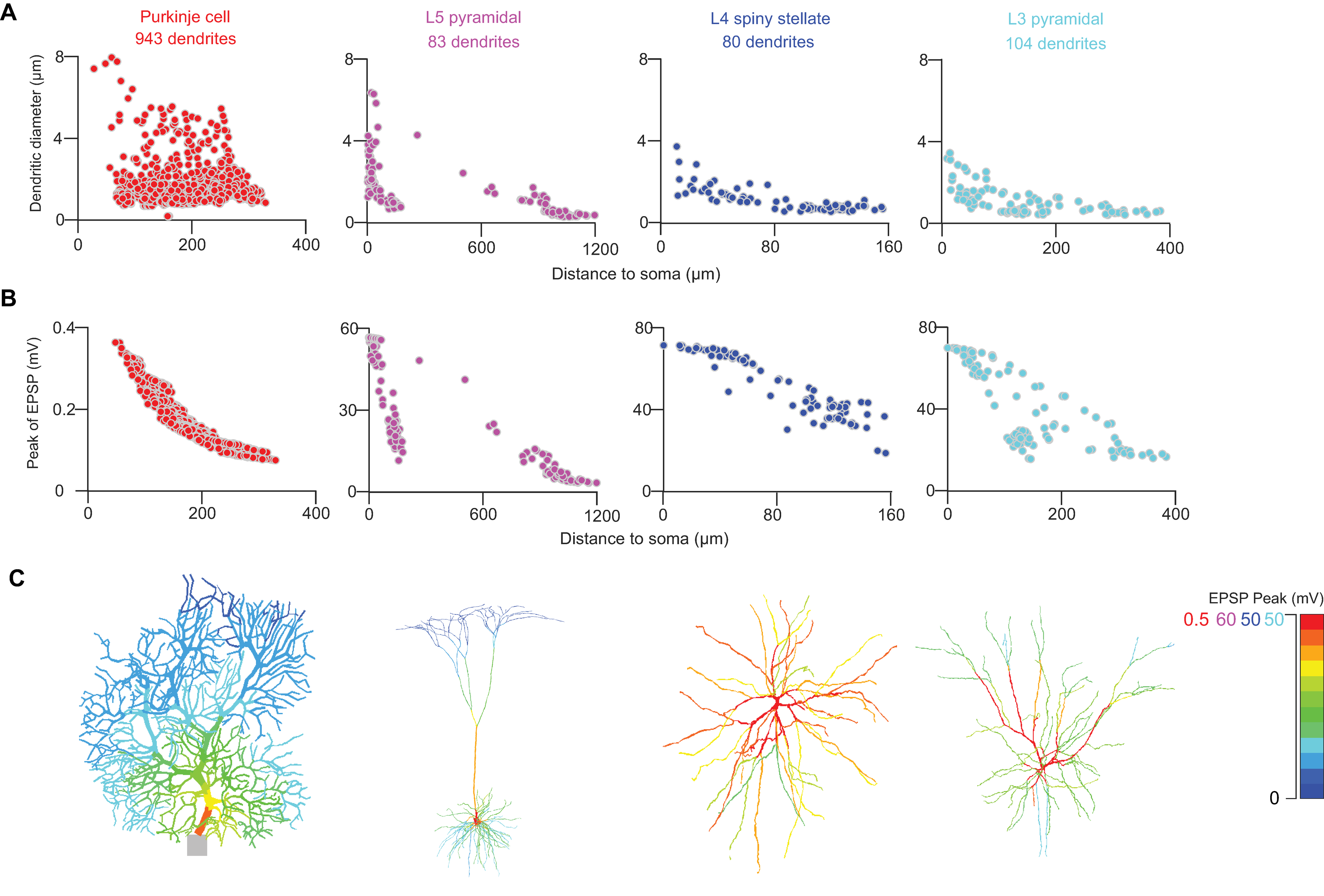

Supplement: S6 Fig — (A) Dendritic diameter vs. dendritic distance to soma. Each data point in the scatter plot represents a single dendrite in the NEURON model. Compared to the Purkinje cell (943 dendrites), three cortical cells have few dendrites (83, 80, and 104 dendrites). (B) The peak amplitude of EPSP (excitatory postsynaptic potential) induced on every single dendrite decays with the distance to soma. (C) The distribution of EPSP peak amplitude over the entire dendritic field. For the scaling bar, max peak values (0.5 mV for PC, 60 mV for L5, 50 mV for L4 and L3) are varying and are colored according to each cell. (TIF) [file pcbi.1011019.s006.tif]
